# MicroRNA-122 as a predictor of HBsAg seroclearance in hepatitis B and C dual infected patients treated with interferon and ribavirin

**DOI:** 10.1038/srep33816

**Published:** 2016-09-26

**Authors:** Yi-Hao Yen, Chao-Min Huang, Kuo-Liang Wei, Jing-Houng Wang, Sheng-Nan Lu, Chuan-Mo Lee, Chao-Hung Hung, Chien-Hung Chen, Po-Lin Tseng, Kuo-Chin Chang, Ming-Chao Tsai, Ming-Tsung Lin, Cheng-Kun Wu, Cheng-Hong Yang, Sin-Hua Moi, Chung-Lung Cho, Tsung-Hui Hu

**Affiliations:** 1 Division of Hepatogastroenterology, Department of Internal Medicine, Kaohsiung Chang Gung Memorial Hospital, Kaohsiung, Taiwan; School of Medicine, College of Medicine, Chang Gung University, Taoyuan, Taiwan; 2 Department of Biological Sciences, National Sun Yat-sen University, Kaohsiung, Taiwan; 3 Division of Gastroenterology and Hepatology, Department of Internal Medicine, Chang Gung Memorial Hospital, Chiayi, Taiwan; 4 Department of Electronic Engineering, National Kaohsiung University of Applied Sciences, Kaohsiung, Taiwan

## Abstract

It has been demonstrated that microRNA-122 (miR-122) plays key roles in the modulation of hepatitis B virus (HBV) replication. This study examined the role of miR-122 in patients with hepatitis C virus (HCV)-HBV dual infection with active hepatitis C who received pegylated interferon-α and ribavirin dual therapy. We enrolled 121 patients with HCV-HBV dual infection after dual therapy. Stored serum was collected before treatment. RT-PCR was used to analyze miR-122. HBsAg seroclearance was noted in 37 (30.1%) cases during a median follow-up period of 5.4 years. miR-122 was significantly lower in HBsAg seroclearance patients than in non-HBsAg seroclearance patients (P < 0.014). Multivariate analysis showed that miR-122 was an independent factor of HBsAg seroclearance (OR: 0.30, 95% CI: 0.09–0.98, P = 0.046). miR-122 was significantly higher in patients who were qHBsAg > 100 IU/mL versus ≤100 IU/mL (P < 0.001). We concluded that in patients with HBV-HCV dual infection with active hepatitis C, miR-122 was associated with HBsAg seroclearance after therapy and qHBsAg level before therapy, indicating that miR-122 plays key roles in modulating HBV replication.

In Taiwan, where patients typically acquire hepatitis B virus (HBV) infection perinatally or during early childhood, HBV and hepatitis C virus (HCV) dual infection is often the result of HCV superinfection. Seroprevalence studies have shown that concurrent HCV infection occurs in approximately 10–15% of patients with chronic HBV infection, although the prevalence may vary between areas and countries^1^,^2^,^3^.

Several studies have shown that HBV-HCV dual infection is associated with faster hepatic fibrosis progression rates, a higher risk of progression to cirrhosis, and a greater risk of hepatic decompensation and hepatocellular carcinoma development compared with HBV or HCV monoinfection^4^,^5^,^6^,^7^,^8^,^9^,^10^,^11^,^12^.

Viral interference is evident between HBV and HCV. HCV mostly exerts a suppressive effect on HBV^1^,^13^,^14^,^15^. Liu *et al*. reported that combination therapy with pegylated interferon-α (Peg-IFN) and ribavirin (RBV) to eradicate HCV is equally effective in patients with HCV monoinfection and in those with chronic HCV-HBV dual infection with active hepatitis C^16^.

MicroRNAs are evolutionarily conserved, small (18–25 ribonucleotides), non-coding RNAs that have important roles in the control of many biological processes, such as cellular development, differentiation, proliferation, apoptosis, and metabolism^17^. The role of miRNAs in modulating the response to hepatotrophic virus infection has been extensively studied.

MicroRNA -122 (miR-122) is specifically expressed and highly abundant in the human liver and is thought to facilitate the replication of HCV RNA^18^. A previous study indicated that serum miR-122 levels were higher in subjects showing a sustained virological response (SVR) to Peg-IFN/RBV therapy in HCV monoinfection^19^. Anti–miR-122 molecules reduced HCV RNA levels in a phase 2a study of G1 patients^20^. miR-122 directly suppresses HBV replication by binding to viral RNA^21^,^22^. Loss of miR-122 expression has also been shown to enhance HBV replication indirectly through cyclin G1-modulated p53 activity^23^. Few studies have examined the role of miR-122 in hepatitis B and C dual infection^24^. Here, we examined the role of miR-122 in patients with chronic HCV and HBV dual infection with active hepatitis C who received Peg-IFN and RBV therapy.

## Patients and Methods

### Patient selection

A total of 121 treatment-naïve chronic HCV-HBV dual-infected patients with active hepatitis C who received dual therapy from 2004 to 2011 and who were followed-up for more than 24 weeks after treatment were enrolled in this study. The end of follow up date is 2015-12-31. Serum hepatitis B surface antigen (HBsAg) levels were measured annually. Ultrasonography and serum α-fetoprotein (AFP) for hepatocellular carcinoma (HCC) surveillance were conducted every 6 months during the follow-up period. There were 59 genotype 1 patients and 62 genotype 2 patients. All patients received response-guided therapy irrespective of their genotype, this therapy was funded by the Bureau of National Health Insurance, Department of Health, Taiwan. The response-guided therapy was as follows: 24 weeks of PEG-IFN and RBV treatment for patients with undetectable HCV RNA at week 4 (defined as rapid virological response); 48 weeks of treatment for patients with detectable HCV RNA at week 4 and undetectable HCV RNA at week 12 (defined as early virological response); and 16 weeks for patients with detectable HCV RNA at week 12. SVR was defined as undetectable HCV RNA at follow-up week 24.

Inclusion criteria were HCV-HBV dual-infected patients with active hepatitis C, defined by seropositivity for both anti-HCV and HBsAg for more than 6 months together with detectable serum HCV-RNA (>50 IU/mL). Patients were excluded if they tested positive for anti-HIV antibody or exhibited other causes of hepatocellular injury (e.g., history of alcoholism, autoimmune hepatitis, primary biliary cirrhosis, or treatment with hepatotoxic drugs). The study was conducted according to the guidelines of the Declaration of Helsinki under the principles of good clinical practice and was approved by local ethics committees. Because of the observational nature of the study, we verbally informed all participants about the study, written informed consent was not required. The subject information was anonymized at collection and anonymized prior to analysis. All methods were performed in accordance with the approved guidelines.

### Quantification of HBsAg and HBV DNA

HBsAg was quantified by using a standard quantitative chemiluminescent microparticle immunoassay (Architect HBsAg, Abbott Diagnostics, Princeton, NJ, USA). The concentration of HBsAg in the specimen was determined using a previously generated Architect HBsAg calibration curve (range, 0.05–250 IU/mL). Serum HBsAg < 0.05 IU/mL was defined as clearance of HBsAg. Samples with serum HBsAg titer >250 IU/mL were diluted to 1:20 and 1:500 with the Architect HBsAg diluent and retested to expand the upper limit of the dynamic range from 250 to 125,000 IU/mL. HBV DNA levels were quantified using the Cobas Taqman assay (Roche Diagnostics, Basel, Switzerland), which has a lower limit of quantification of 60 copies/mL (12 IU/mL) and a linear range of upper detection limit of 6.4 × 10^8 ^copies/mL (1.3 × 10^8 ^IU/mL). For results exceeding the upper detection limit, HBV DNA was remeasured after 100,000-fold dilution.

### HCV RNA and genotyping

Qualitative detection of HCV RNA was performed using a standardized reverse transcription polymerase chain reaction (RT-PCR) assay (Amplicor, Roche Diagnostics), using biotinylated primers for the 5′-non-coding region. The lowest detection limit for this assay was 15 IU/mL. HCV genotyping was performed in a reverse hybridization assay (Inno-LiPA^TM^ HCV II; Innogenetics N.V., Gent, Belgium) by using HCV-Amplicor products.

### Blood sampling

Peripheral blood (PB) was collected before therapy from each individual and placed directly into serum tubes. Ethylenediaminetetraacetic acid (EDTA)-PB samples were temporarily preserved at 4 °C after collection and processed within 4 h of collection. PB samples were centrifuged at 3500 × *g* for 15 min to obtain the plasma, and then ammonium chloride lysis buffer (10 mM NH_4_Cl, 10 mM KHCO_3_, 0.1 mM EDTA) was used to deplete red blood cells from the PB for total leukocyte isolation. The separated plasma samples were preserved at −20 °C until small RNA extraction.

### Small RNA isolation from plasma

Isolation of small RNA from 500 μL of plasma was performed using a mirVana™ PARIS™ kit (Ambion, Foster City, CA, USA) according to the manufacturer’s protocols. Isolated small RNA was eluted in 100 μL nuclease-free water. cDNAs were storage at −20 °C in RNase free water.

### Real-time quantitative reverse transcription-PCR analysis of miRNAs

Mature microRNA expression was quantified by real-time quantitative RT-PCR using TaqMan^®^ microRNA assays according to the manufacturer’s protocols (Applied Biosystems, Foster City, CA, USA), which included two steps: the RT reaction and the TaqMan real-time PCR assay. Briefly, RT reactions were performed using 10 ng of total RNA, 50 nM stem-loop microRNA-specific RT primers, 1 × RT buffer, 0.25 mM of dNTPs, 3.33 U/μL MultiScribe RTase, and 0.25 U/μL RNase inhibitor. The reaction mixture was incubated for 30 min at 16 °C and 30 min at 42 °C, followed by 5 min incubation at 85 °C to inactivate the RTase enzyme. RT products were subjected to microRNA expression analyses for real-time quantitative PCR in a 20-μL final volume containing 2 μL of RT product, 1 μL of 20× TaqMan micro-RNA Assay (Applied Biosystems), and 10 μL of 2× TaqMan Universal PCR Master Mix (Applied Biosystems). The PCR cycling parameters were 95 °C for 15 s followed by 60 °C for 30 s for 40 cycles. Quantitative real-time PCR was performed in triplicate. The cycle threshold (*C*_T_ value) is defined as the number of cycles required for the fluorescent signal to cross the threshold in qPCR and is inversely correlated with the miRNA level. As there is currently no consensus on a suitable normalization control for serum miRNA profiling, and miR-16 has been shown to serve as a stable reference normalization control^25^, we used it as normalization control in the present study.The relative expression levels of serum miR-122 were calculated using a ΔCt method where, ΔCt = Ct(miR-16)–Ct(miR-122).

### Genetic variation of interleukin-28B (IL-28B) polymorphism

A single-nucleotide polymorphism of IL-28B was determined by direct sequencing using TaqMan Pre-Designed SNP Genotyping Assays (PE Applied Biosystems) as recommended by the manufacturer.

### Statistical analysis

Means and standard deviations were used to describe the distribution of continuous variables. Independent *t*-tests were used to compare continuous variables. Univariate logistic regression analysis was used to identify independent factors that may influence HBsAg seroclearance, HCC development, and HCV SVR. The significant clinical factors in univariate analysis (P < 0.05) were included as covariate in multivariate logistic regression models. The cumulative probabilities of HBsAg seroclearance and HCC development were analyzed by the Kaplan-Meier curve method with the log-rank test. In all analyses, a p-value < 0.05 is considered statistically significant. All statistical analyses were performed using STATA version 11.1 (StataCorp, College Station, TX, USA).

## Results

### Patient demographics

Table 1 shows the baseline data of the 121 patients. The median follow-up period was 5.43 (3.42–7.00) years after the end of treatment. Five (6.9%) patients were seropositive for HBeAg. The median HBV DNA was 55 IU/mL. The median HCV RNA level was 346508 IU/mL. Ninety-one (75%) patients achieved HCV SVR after combination therapy. During the median of 5.4 years of follow-up, 24 patients (19.8%) showed HCC development and 37 cases (30.1%) showed serum HBsAg seroclearance.

### Correlation between miR-122 and clinical parameters

The expression levels of miR-122 were not significantly different between sex, liver cirrhosis, aspartate aminotransferase (AST), alanine aminotransferase (ALT), HCV genotype, IL-28B genotype, HBeAg status and HBV DNA levels (Table 2). However, miR122 was significantly higher in qHBsAg > 100 IU/mL versus qHBsAg ≤ 100 IU/mL (P < 0.001) and HCV RNA ≤ 4 × 10^5 ^IU/ml versus HCV RNA > 4 × 10^5 ^IU/ml (P = 0.038).

### Association between clinico-biochemical factors and HCV SVR

There were 59 HCV genotype 1 patients (Table 3). Forty-four (75%) of these patients achieved SVR. There were 62 genotype 2 patients (Table 4), and 47 (76%) of these patients achieved SVR. miR-122 was not significantly different in SVR versus non-SVR in genotype 1 and 2 patients. Independent factors associated with HCV SVR were male (OR: 5.91, 95% CI: 1.46–23.9, P = 0.013) in genotype 1 patients, and AFP > 20ng/ml (OR:0.11, 95% CI: 0.02–0.78, P = 0.027), liver cirrhosis (OR:0.22, 95% CI: 0.06–0.86, P = 0.029) in genotype 2 patients.

### Association between clinic-biochemical factors and HBsAg seroclearance

**Figure 1:**
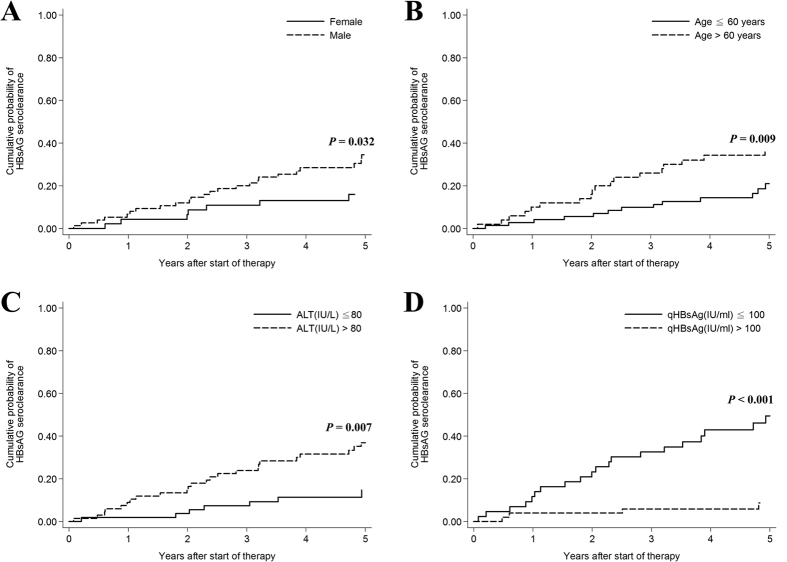
Kaplan–Meier analysis of independent factors associated with HBsAg seroclearance. qHBsAg: quantitative hepatitis B surface antigen.

**Figure 2:**
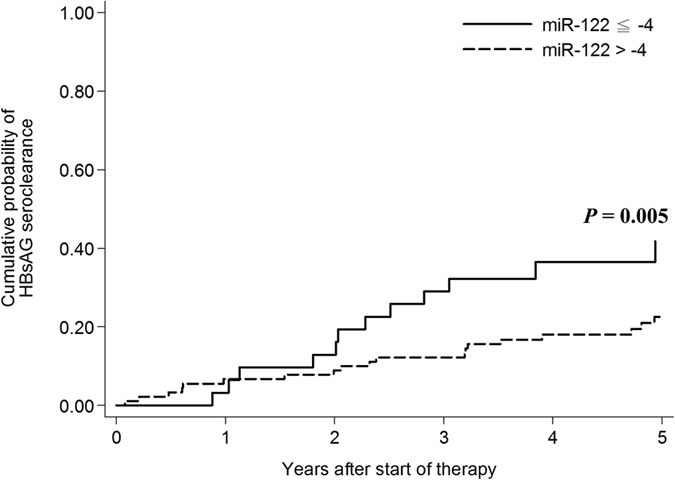
Kaplan–Meier analysis of independent factors associated with HBsAg seroclearance. The relative expression levels of serum miR-122 were calculated using a ΔCt method where, ΔCt = Ct(miR-16) – Ct(miR-122). The cycle threshold (*C*_T_ value) is defined as the number of cycles required for the fluorescent signal to cross the threshold in qPCR, inversely correlates with the miRNA level.

HBsAg seroclearance was noted in 37 (31%) patients during follow-up. The 5-year cumulative probability of HBsAg seroclearance was 27.84% (95% CI = 20.31–37.44), with an average annual rate of 5.57%. Univariate analysis (Table 5) showed that male patients (P = 0.043), age > 60 years (P = 0.003), ALT > 80 IU/mL (P = 0.001), miR-122 ≤ −4(P = 0.014) and qHBsAg < 100 IU/mL (P < 0.001) were associated with HBsAg seroclearance. Multivariate analysis showed that male (OR = 4.43, 95% CI: 1.35–14.58, P = 0.014), age > 60 years (OR = 4.33, 95% CI: 1.40–13.45, P = 0.011), ALT > 80IU/ml (OR:4.24, 95% CI: 1.32–13.62, P = 0.015), miR-122> −4 (OR = 0.30, 95% CI: 0.09–0.98, P = 0.046), and qHBsAg > 100 IU/ml (OR = 0.22, 95% CI: 0.06–0.81, P = 0.023) were independently associated with HBsAg seroclearance. Kaplan–Meier analysis of independent factors associated with HBsAg seroclearance were shown in Figs 1 and 2.

### Association between clinic-biochemical factors and HCC development

Twenty-four (19.8%) patients developed HCC during follow-up. The 5-year cumulative probability of HCC development was 4.27% (95% CI = 1.60–11.12), with an average annual rate of 0.85%. Univariate analysis (Table 6) showed that the age > 60 years (P = 0.022), AFP > 20 ng/mL (P = 0.018), liver cirrhosis (P < 0.001), HBV DNA > 2000 IU/mL (P = 0.04), and non-HCV SVR (P = 0.01) were higher in HCC patients compared to that in non-HCC patients. Multivariate analysis showed that liver cirrhosis (OR = 7.31, 95% CI: 2.22–24.06, P = 0.001) and HBV DNA >2000 IU/ml (OR = 6.29, 95% CI: 1.44–27.43, P = 0.014) were independent factors correlated with HCC development.

## Discussion

In this study, we found that in patients dually infected with chronic HBV-HCV and active hepatitis C, the baseline miR-122 level was correlated with qHBsAg levels at baseline and could be used to predict HBsAg seroclearance after Peg-IFN and RBV treatment. Previous studies demonstrated that the serum miR-122 level may predict HCV SVR in patients with genotype 2 HCV monoinfection^19^. In this study, we found that miR-122 was not associated with SVR according to multivariate analysis. Since the number of cases in this study was small, including only 62 genotype 2 dual-infected patients, further studies of a larger number of subjects is needed to explore the association between the miR-122 level and HCV SVR.

HBs antigen and HBV DNA levels were quantified as surrogate parameters for HBV replication and translation. Previous studies evaluating chronic hepatitis B monoinfected patients revealed a positive correlation between miR-122 expression and HBV DNA^26^,^27^,^28^,^29^, miR-122 was positively correlated to the HBsAg titer^27^,^28^,^29^, and miR-122 levels were significantly higher in HBeAg-positive patients than in HBeAg-negative patients^28^. These findings obtained from HBV monoinfected patients were similar to those from hepatitis B and C dual-infected patients in our study. Although the reason for the higher levels of miR-122 in patients with active HBV replication is unclear, the innate immune response in liver cells against HBV replication may induce higher expression of miR-122, which can be reflected in serum levels^28^. The other explanation is that miR-122 may passively leak from damaged or dying cells because of apoptosis or necrosis. HBV-induced cell damage involves a combination of HBV-induced apoptotic and T cell–induced cell death^30^. Circulating miR-122 has been described as a sensitive marker of hepatocyte damage, and thus may originate from dying cells^31^.

Studies related to serum microRNAs in chronic hepatitis B- or C-infected patients have been widely reported. However, few studies have examined serum microRNAs in chronic dual HBV-HCV-infected patients^24^. Cheng *et al*. evaluated 76 patients with HBV/HCV dual-infection who received Peg-IFN-based treatment. In multivariate analysis, the serum miR-122 level was positively correlated with serum qHBsAg. A high baseline miR-122 level was positively correlated with a greater reduction in the post-treatment serum qHBsAg level^24^. In our study, we also found that miR122 was significantly higher in patients with qHBsAg > 100 IU/mL. Cheng *et al*. also observed HBsAg seroclearance in 14 patients after a 6-month follow-up post-PEG-IFN-based treatment. The miR-122 level was not correlated with HBsAg seroclearance^24^. In contrast, we found that miR-122 was independently correlated with HBsAg seroclearance. The discrepancy may be that Cheng *et al*. only evaluated HBsAg seroclearance at 6 months after treatment. However, we determined HBsAg seroclearance annually after treatment over a longer follow-up (median 5.4 years).

HBsAg seroclearance typically confers a favorable outcome and is the optimal treatment goal. However, HBsAg seroclearance is very rarely observed in HBV mono-infected patients receiving currently available antiviral agents, with an annual rate of 2.4–3.2% with IFN or Peg-IFN therapy and only 1% with nucleoside/nucleotide analogues^32^,^33^. Previous studies showed that 25.6–30% of HBV/HCV dually infected patients treated with PEG-IFN/RBV combination therapy achieved HBsAg seroclearance during the 5-year follow-up period^34^,^35^, which agrees with the results of our study. This higher rate of HBsAg seroclearance suggests that viral interaction between HBV and HCV influences the rate of HBsAg seroclearance.

Factors related to HBsAg seroclearance in hepatitis B and C dual infection include liver cirrhosis and HBV DNA negativity at 1 year after end-of-treatment^34^, low baseline serum HBV DNA^34^, and low baseline HBsAg levels^16^,^35^,^36^. In our study, multivariate analysis showed that male, age > 60 years, ALT > 80 IU/L, qHBsAg ≤ 100 IU/ml and low miR-122 levels were independently correlated with HBsAg seroclearance. The possible mechanism of this novel finding is unclear, but miR-122 may play a key role in modulating HBV replication in the viral interaction with HCV^21^,^22^,^23^.

Although previous studies showed that miR-122 was associated with HCC^37^,^38^,^39^, miR-122 was not associated with HCC development in our study.

In conclusion, for patients with chronic HBV-HCV dual infection and active hepatitis C, baseline miR-122 was correlated with baseline qHBsAg level and independently predicted HBsAg seroclearance after Peg-IFN and RBV treatment. Further long-term and large-scale studies are warranted.

## Additional Information

**How to cite this article**: Yen, Y.-H. *et al*. MicroRNA-122 as a predictor of HBsAg seroclearance in hepatitis B and C dual infected patients treated with interferon and ribavirin. *Sci. Rep.* **6**, 33816; doi: 10.1038/srep33816 (2016).

## Figures and Tables

**Table 1:** Baseline characterisitic of all patients.

Variables	
Age (years)	56.9 ± 10.9
Male	75 (62.0%)
AST (IU/L)	82.9 ± 55.1
ALT (IU/L)	151.4 ± 234.1
bilirubin (mg/dL)	0.9 ± 0.3
Liver cirrhosis	32 (26.5%)
HCV SVR	91 (75.2%)
HCV Genotype 1	59 (48.8%)
HCV Genotype 2	62 (51.2%)
HCV RNA (IU/ml)	346508 (41180–1201995)
miR122	−2.0 ± 3.3
rs12979860 CC	102 (84.3%)
rs12980275AA	104 (85.95%)
rs8099917TT	104 (85.95%)
HBeAg positive	8 (6.6%)
HBV DNA (IU/ml)	55 (0–1399)
qHBsAg (IU/ml)	139.0 (8.1–1304.2)
BMI(kg/m2)	24.6 ± 3.9
Platelet <15(1000/μL)	67 (55.4%)
AFP>20 (ng/mL)	13 (10.7%)
DM	23 (19.0%)
Treatment duration	
24 weeks	81 (66.9%)
48 weeks	40 (33.1%)
HCC	24 (19.8%)
HBsAg seroclearance	37 (30.6%)
Follow up duration (years)	5.4 (3.4–7.0)

Data were expressed as mean  ±  SD or median (interquantile). AST, Aspartate Aminotransferase; ALT, Alanine Aminotransferase; HCV, hepatitis C virus; SVR, sustained virological response; HBeAg, hepatitis B e antigen, HBV, hepatitis B virus; qHBsAg, quantitative hepatitis B surface antigen; BMI, body mass index; AFP, α-fetoprotein; DM, diabetes mellitus; HCC, hepatocellular carcinoma; The relative expression levels of serum miR-122 were calculated using a ΔCt method where, ΔCt = Ct(miR-16) – Ct(miR-122). The cycle threshold (*C*_T_ value) is defined as the number of cycles required for the fluorescent signal to cross the threshold in qPCR, inversely correlates with the miRNA level.

**Table 2:** Correlation of serum miR-122 levels and clinical parameters.

Variables	miR122	*P* value
Male	−2.13 ± 3.51	0.566
Female	−1.76 ± 3.04	
Age > 60 years	−2.05 ± 3.57	0.868
Age ≤60 years	−1.95 ± 3.18	
Liver cirrhosis	−1.84 ± 3.31	0.765
Non-liver cirrhosis	−2.04 ± 3.36	
ALT>80 U/L	−1.74 ± 3.00	0.365
ALT≤80 U/L	−2.30 ± 3.72	
Genotype 1	−2.25 ± 3.51	0.403
Genotype 2	−1.74 ± 3.17	
HCV RNA> 4 × 10^5 ^IU/ml	−3.52 ± 3.07	0.038
HCV RNA≤ 4 × 10^5 ^IU/ml	−1.87 ± 3.73	
rs12979860 CC	−1.85 ± 3.08	0.298
rs12979860 non-CC	−2.72 ± 4.50	
rs 12980275 AA	−1.96 ± 3.16	0.844
rs 12980275 non-AA	−2.14 ± 4.36	
rs8099917 TT	−1.96 ± 3.16	0.844
rs8099917 non-TT	−2.14 ± 4.36	
HBeAg positive	−0.07 ± 1.86	0.087
HBeAg negative	−2.08 ± 3.25	
HBV DNA > 2000 IU/ml	−0.57 ± 2.43	0.079
HBV DNA ≤ 2000 IU/ml	−2.02 ± 3.29	
qHBsAg>100 IU/ml	−0.37 ± 2.53	<0.001
qHBsAg≤100 IU/ml	−3.53 ± 2.75	

AST, Aspartate Aminotransferase; ALT, Alanine Aminotransferase; HCV, hepatitis C virus; HBeAg, hepatitis B e antigen, HBV, hepatitis B virus; qHBsAg, quantitative hepatitis B surface antigen; The relative expression levels of serum miR-122 were calculated using a ΔCt method where, ΔCt = Ct(miR-16) – Ct(miR-122). The cycle threshold (*C*_T_ value) is defined as the number of cycles required for the fluorescent signal to cross the threshold in qPCR, inversely correlates with the miRNA level.

**Table 3:** Univariate and multivariate analysis of factors associated with sustained virological response in HCV genotype 1 patients.

Variables	comparison	OR	Univariate analysis	*P*	OR	Multivariate analysis	*P*
			95% CI			95% CI	
Sex	Male vs female	6.56	1.76–24.42	0.005	5.91	1.46–23.9	0.013
Age (years)	>60 vs ≤60	0.33	0.10–1.10	0.072			
BMI(Kg/m^2^)	>24 vs ≤24	1.05	0.29–3.77	0.943			
DM	Yes vs no	0.20	0.05–0.88	0.034	0.28	0.05–1.65	0.159
AST (IU/L)	>80 vs ≤80	0.40	0.11–1.41	0.153			
ALT(IU/L)	>80 vs ≤80	0.80	0.25–2.59	0.708			
Platelet (1000/μL)	>15 vs ≤15	1.49	0.44–5.05	0.525			
AFP(ng/mL)	>20 vs ≤20	0.83	0.14–4.82	0.839			
HCV RNA (IU/ml)	> 4 × 10^5^ vs ≤4 × 10^5^	0.33	0.06–1.84	0.204			
Liver cirrhosis	Yes vs no	0.23	0.06–0.79	0.019	0.31	0.07–1.28	0.106
rs12979860	CC vs non-CC	2.84	0.65–12.40	0.166			
rs12980275	AA vs non-AA	4.97	0.97–25.57	0.055			
rs8099917	TT vs non-TT	4.97	0.97–25.57	0.055			
miR122	Mean (SD)	0.97	0.82–1.15	0.735			
HBe Ag	Positive vs negative	0.31	0.02–5.30	0.418			
HBV DNA (IU/ml)	>2000 vs ≤2000	0.44	0.10–2.00	0.286			
qHBsAg (IU/ml)	>100 vs ≤100	0.89	0.21–3.72	0.872			
Treatment duration (weeks)	48 vs 24	1.83	0.54-–6.22	0.336			

BMI, body mass index; DM, diabetes mellitus; AST, Aspartate Aminotransferase; ALT, Alanine Aminotransferase; AFP, alpha-feto protein, HCV, hepatitis C virus; HBeAg, hepatitis B e antigen, HBV, hepatitis B virus; qHBsAg, quantitative hepatitis B surface antigen; The relative expression levels of serum miR-122 were calculated using a ΔCt method where, ΔCt = Ct(miR-16) – Ct(miR-122). The cycle threshold (*C*_T_ value) is defined as the number of cycles required for the fluorescent signal to cross the threshold in qPCR, inversely correlates with the miRNA level.

**Table 4:** Univariate and multivariate analysis of factors associated with sustained virological response in HCV genotype 2 patients.

Variables	comparison	OR	Univariate analysis	*P*	OR	Multivariate analysis	*P*
			95% CI			95% CI	
Sex	Male vs female	1.87	0.57–6.11	0.302			
Age (years)	>60 vs ≤60	0.54	0.17–1.76	0.305			
BMI(Kg/m^2^)	>24 vs ≤24	1.63	0.5–5.34	0.415			
DM	Yes vs no	0.31	0.09–1.11	0.072			
AST (IU/L)	>80 vs ≤80	1.57	0.48–5.10	0.457			
ALT(IU/L)	>80 vs ≤80	3.20	0.96–10.64	0.058			
Platelet (1000/μL)	>15 vs ≤15	0.98	0.81–10.88	0.099			
AFP(ng/mL)	>20 vs ≤20	0.12	0.02–0.76	0.024	0.11	0.02–0.78	0.027
HCV RNA (IU/ml)	> 4 × 10^5^ vs ≤4 × 10^5^	0.33	0.08–1.41	0.136			
Liver cirrhosis	Yes vs no	0.23	0.07–0.83	0.025	0.22	0.06–0.86	0.029
rs12979860	CC vs non-CC	0.75	0.14–3.99	0.736			
rs12980275	AA vs non-AA	0.75	0.14–3.99	0.736			
rs8099917	TT vs non-TT	0.75	0.14–3.99	0.736			
miR122	Mean (SD)	1.20	0.99–1.46	0.069			
HBe Ag	Positive vs negative	–	–	–			
HBV DNA (IU/ml)	>2000 vs ≤2000	0.83	0.14–4.91	0.835			
qHBsAg (IU/ml)	>100 vs ≤100	2.44	0.53–11.17	0.249			
Treatment duration (weeks)	48 vs 24	1.22	0.29–5.13	0.784			

BMI, body mass index; DM, diabetes mellitus; AST, Aspartate Aminotransferase; ALT, Alanine Aminotransferase; AFP, alpha-feto protein, HCV, hepatitis C virus; HBeAg, hepatitis B e antigen, HBV, hepatitis B virus; qHBsAg, quantitative hepatitis B surface antigen; The relative expression levels of serum miR-122 were calculated using a ΔCt method where, ΔCt = Ct(miR-16) – Ct(miR-122). The cycle threshold (*C*_T_ value) is defined as the number of cycles required for the fluorescent signal to cross the threshold in qPCR, inversely correlates with the miRNA level.

**Table 5:** Univariate and multivariate analysis of factors associated with hepatitis B surface antigen seroclearance after antiviral therapy.

Variables	comparison	OR	Univariate analysis	*P*	OR	Multivariate analysis	*P*
			95% CI			95% CI	
sex	Male vs female	2.45	1.03–5.82	0.043	4.43	1.35–14.58	0.014
Age (years)	>60 vs ≤60	3.47	1.55–7.77	0.003	4.33	1.40–13.45	0.011
BMI(Kg/m^2^)	>24 vs ≤24	1.92	0.86–4.28	0.111			
DM	Yes vs no	1.27	0.49–3.32	0.627			
AST (IU/L)	>80 vs ≤80	2.11	0.95–4.69	0.066			
ALT(IU/L)	>80 vs ≤80	4.39	1.80–10.72	0.001	4.24	1.32–13.62	0.015
Platelet (1000/μL)	>15 vs ≤15	0.90	0.41–2.00	0.803			
AFP(ng/mL)	>20 vs ≤20	3.03	0.94–9.76	0.063			
HCV RNA (IU/ml)	>4 × 10^5^ vs ≤4 × 10^5^	0.86	0.30–2.46	0.772			
Liver cirrhosis	Yes vs no	1.27	0.54–3.00	0.587			
rs12979860	CC vs non-CC	0.55	0.20–1.50	0.239			
miR122	>−4 vs ≤−4	0.35	0.15–0.81	0.014	0.30	0.09–0.98	0.046
HBV DNA (IU/ml)	>2000 vs ≤ 2000	0.85	0.25–2.93	0.797			
qHBsAg (IU/ml)	>100 vs ≤100	0.10	0.03–0.31	<0.001	0.22	0.06–0.81	0.023
Treatment duration (weeks)	48 vs 24	1.14	0.51–2.58	0.747			
HCV SVR	Yes vs no	0.84	0.35–2.04	0.706			
HCV genotype	2 vs 1	0.86	0.40–1.87	0.705			

BMI, body mass index; DM, diabetes mellitus; AST, Aspartate Aminotransferase; ALT, Alanine Aminotransferase; AFP, alpha-feto protein, HCV, hepatitis C virus; HBeAg, hepatitis B e antigen, HBV, hepatitis B virus; qHBsAg, quantitative hepatitis B surface antigen; The relative expression levels of serum miR-122 were calculated using a ΔCt method where, ΔCt = Ct(miR-16) – Ct(miR-122). The cycle threshold (*C*_T_ value) is defined as the number of cycles required for the fluorescent signal to cross the threshold in qPCR, inversely correlates with the miRNA level.

**Table 6:** Univariate and multivariate analysis of factors associated with hepatocellular carcinoma development after antiviral therapy.

Variables	comparison	OR	Univariate analysis	*P*	OR	Multivariate analysis	*P*
			95% CI			95% CI	
sex	Male vs female	1.63	0.62–4.30	0.321			
Age (years)	>60 vs ≤60	2.95	1.17–7.44	0.022	1.63	0.51–5.23	0.415
BMI(Kg/m^2^)	>24 vs ≤24	0.72	0.29–1.81	0.486			
DM	Yes vs no	2.08	0.74–5.84	0.162			
AST (IU/L)	>80 vs ≤80	2.36	0.95–5.86	0.063			
ALT(IU/L)	>80 vs ≤80	1.16	0.47–2.87	0.745			
Platelet (1000/μL)	>15 vs ≤15	0.56	0.23–1.38	0.207			
AFP(ng/mL)	>20 vs ≤20	4.29	1.29–14.26	0.018	3.22	0.72–14.33	0.125
HCV RNA (IU/ml)	>4 × 10^5^ vs ≤4 × 10^5^	0.53	0.17–1.61	0.261			
Liver cirrhosis	Yes vs no	7.84	2.95–20.86	<0.001	7.31	2.22–24.06	0.001
rs12979860	CC vs non-CC	2.34	0.50–10.90	0.280			
miR122	Mean (SD)	1.03	0.90–1.18	0.671			
HBeAg	Positive vs negative	0.56	0.07–4.78	0.595			
HBV DNA (IU/ml)	>2000 vs ≤2000	3.33	1.05–10.49	0.040	6.29	1.44–27.43	0.014
qHBsAg (IU/ml)	>100 vs ≤100	0.94	0.33–2.69	0.904			
HBsAg seroclearance	Yes vs no	1.17	0.45–3.04	0.744			
HCV SVR	Yes vs no	0.29	0.11–0.74	0.010	0.63	0.20–1.96	0.423
HCV genotype	2 vs 1	1.16	0.47–2.84	0.749			

BMI, body mass index; DM, diabetes mellitus; AST, Aspartate Aminotransferase; ALT, Alanine Aminotransferase; AFP, alpha-feto protein, HCV, hepatitis C virus; HBeAg, hepatitis B e antigen, HBV, hepatitis B virus; qHBsAg, quantitative hepatitis B surface antigen; The relative expression levels of serum miR-122 were calculated using a ΔCt method where, ΔCt = Ct(miR-16) – Ct(miR-122). The cycle threshold (*C*_T_ value) is defined as the number of cycles required for the fluorescent signal to cross the threshold in qPCR, inversely correlates with the miRNA level.
